# Generation of a Biomimetic Substitute of the Corneal Limbus Using Decellularized Scaffolds

**DOI:** 10.3390/pharmaceutics13101718

**Published:** 2021-10-17

**Authors:** David Sánchez-Porras, Manuel Caro-Magdaleno, Carmen González-Gallardo, Óscar Darío García-García, Ingrid Garzón, Víctor Carriel, Fernando Campos, Miguel Alaminos

**Affiliations:** 1 Department of Histology and Tissue Engineering Group, Faculty of Medicine, Universidad de Granada and Instituto de Investigación Biosanitaria ibs.GRANADA, E18016 Granada, Spain; david.s.p.94@gmail.com (D.S.-P.); garciagarciaoscar2b@gmail.com (Ó.D.G.-G.); igarzon@ugr.es (I.G.); vcarriel@ugr.es (V.C.); 2 Division of Ophthalmology, University Hospital Virgen Macarena, Universidad de Sevilla, E41009 Seville, Spain; drmanuelcaro@gmail.com; 3 Division of Ophthalmology, University Hospital San Cecilio, E18016 Granada, Spain; carmengonzalez23283@hotmail.com; 4 Doctoral Programme in Biomedicine, Escuela Internacional de Posgrado, Universidad de Granada, E18071 Granada, Spain

**Keywords:** corneal limbus, decellularized xenograft, recellularization, mesenchymal stem cells

## Abstract

Patients with severe limbal damage and limbal stem cell deficiency are a therapeutic challenge. We evaluated four decellularization protocols applied to the full-thickness and half-thickness porcine limbus, and we used two cell types to recellularize the decellularized limbi. The results demonstrated that all protocols achieved efficient decellularization. However, the method that best preserved the transparency and composition of the limbus extracellular matrix was the use of 0.1% SDS applied to the half-thickness limbus. Recellularization with the limbal epithelial cell line SIRC and human adipose-derived mesenchymal stem cells (hADSCs) was able to generate a stratified epithelium able to express the limbal markers p63, pancytokeratin, and crystallin Z from day 7 in the case of SIRC and after 14–21 days of induction when hADSCs were used. Laminin and collagen IV expression was detected at the basal lamina of both cell types at days 14 and 21 of follow-up. Compared with control native limbi, tissues recellularized with SIRC showed adequate picrosirius red and alcian blue staining intensity, whereas limbi containing hADSCs showed normal collagen staining intensity. These preliminary results suggested that the limbal substitutes generated in this work share important similarities with the native limbus and could be potentially useful in the future.

## 1. Introduction

Numerous diseases, including trauma, infections, congenital malformations, degeneration, and other conditions, may affect the transparency of the human cornea and cause blindness [1]. Cornea transplantation or keratoplasty is the gold-standard treatment for severe corneal diseases. However, keratoplasty is subjected to donor shortage [2], and is contraindicated in patients with severe limbal damage and limbal stem cell deficiency (LSCD) [3]. Patients affected by severe LSCD typically show corneal conjunctivalization and neovascularization, and the management of this condition is challenging [4].

In cases with unilateral disease, LSCD can be treated by transplanting autologous limbal tissue from a healthy eye to a damaged eye [5]. Autologous grafts are free from the risk of immune rejection, but are not available in bilateral cases and can potentially compromise the healthy donor eye, resulting in LSCD [6]. If an autologous transplant is not available, patients can be treated with allogeneic limbal grafts obtained from cadaveric or living donors [5,6]. Allogeneic grafts are also subjected to important concerns, such as the risk of immune rejection [5] and graft survival [7]. The lack of a fully safe and efficient treatment makes necessary the search of therapeutic alternatives.

In this regard, the development of cell culture methods allowing cell isolation and expansion is a major advance in the treatment of LSCD [6]. Using small limbal tissue biopsies, current technology allows the generation of limbal stem cell populations that can be implanted in patients with LSCD [8]. Cultured limbal stem cells can be grafted as isolated cells or by using different types of carriers and biomaterials such as the human amniotic membrane, fibrin, collagen, or synthetic biopolymers [9,10,11]. Alternative approaches such as the use of cultured oral mucosa keratinocytes have also been proposed for LSCD treatment [12]. Although promising, the clinical usefulness of most of these treatments should still be demonstrated.

Development of novel tissue engineering technologies allowed the design and construction of human organs that could replace damaged tissues [13]. Bioartificial tissues and organs can be generated using different methods and techniques. Two of the most promising methods are organ bioprinting [14,15] and scaffolds seeded with living cells [16,17]. On the one hand, bioprinting offers the possibility of fabricating complex constructs in which cells and biomaterials can be precisely deposited in a specific 3D structure. However, the fine structure of the human cornea and corneal limbus is very complex, and alternative approaches including the development of transparent bio-inks and complex design protocols are in need to generate efficient limbal substitutes using bioprinting [14,15]. On the other hand, cell-seeded scaffolds have been extensively used in cornea tissue engineering. In general, these methods make use of different types of biomaterials that can be prepared in the laboratory and subsequently seeded with living cells to generate a tissue substitute [16,17]. In the case of the cornea, several models of bioartificial corneas have been developed [18,19], and some of these models have been clinically evaluated [20,21,22]. Again, these techniques need to be significantly improved to allow the efficient reproduction of the delicate histoarchitecture of these tissues.

One of the possible biofabrication alternatives used in cornea tissue engineering is xenograft decellularization. Decellularized natural tissues have the advantage of faithfully reproducing the native extracellular matrix (ECM) [23]. Although a number of works have focused on the development of decellularization protocols applied to the native cornea, a fully efficient protocol able to preserve all ECM components is in need. In general, cornea xenografts can be decellularized by using chemical, physical, and biological methods [24]. Chemical protocols typically use different types of detergents such as sodium dodecyl sulfate (SDS) and Triton X-100, although ethylenediaminetetraacetic acid (EDTA) and hypertonic salts have also been used [25,26]. Physical methods are mostly based on freeze-thawing, osmotic pressure, and lyophilization, whereas biological protocols make use of enzymes such as trypsin, DNAse, and RNAse [24,27]. Although little clinical experience is available for the use of decellularized corneal xenografts, some preliminary clinical trials have pointed out the biosafety and functionality of decellularized porcine corneas in patients subjected to lamellar keratoplasty [26,27,28,29].

Regarding the corneal limbus, very few works focused on the optimization of decellularization protocols specifically applied to this structure [30]. Allocated at the transition between the cornea and the sclera, the corneal limbus plays a key role in maintaining corneal physiology, and its integrity and function are crucial for a normal corneal homeostasis [31]. The three-dimensional structure of the limbus is very complex. The crypts of the limbus form specific pocket-like structures containing fibrovascular Vogt palisades that make up a fundamental micro-niche that houses and supports the limbal stem cells [32].

Unfortunately, the complex structure of the limbus is very difficult to reproduce in the laboratory using standard tissue engineering protocols. However, the use of decellularization methods applied to the native limbi provides the specific morphology, structure, and protein composition of the corneal limbus [30], and offers the opportunity of obtaining adequate limbal scaffolds for use in tissue engineering. In fact, some preliminary reports using SDS and NaCl decellularization protocols have described the efficient generation of limbal substitutes for use in regenerative medicine [11,28,30].

On the other hand, the search for alternative sources of extraocular cells free from the drawbacks and limitations associated with autologous limbal cells used in the treatment of LSCD is in need [33]. In this sense, a possible alternative is the use of human adipose-derived mesenchymal stem cells (hADSCs), which have previously been shown to have differentiation potential to several types of corneal cells both ex vivo and in vivo [34].

In the present preliminary work, we evaluated several decellularization methods applied to the corneal limbus, and we generated recellularized limbal xenografts for future use in patients with limbal damage using two different cell sources.

## 2. Materials and Methods

### 2.1. Obtaining Decellularized Xenografts from Native Limbi

**Figure 1:**
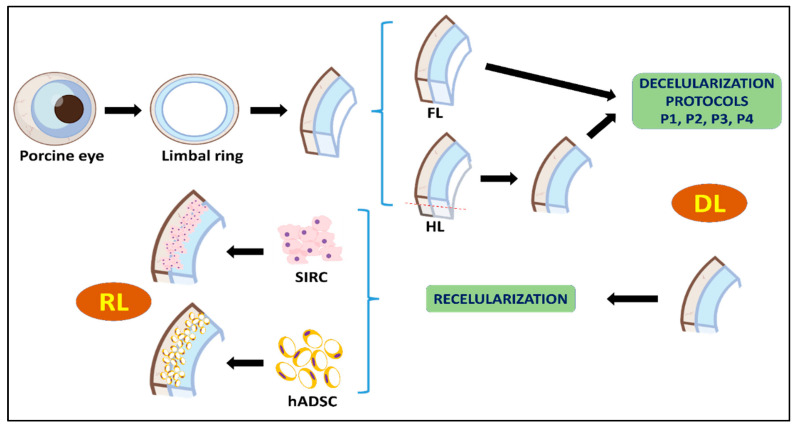
Schematic representation of the study protocol used in the present work. Fl: full-thickness limbus; HL: half-thickness limbus; DL: decellularized limbus; RL: recellularized limbus.

The study protocol is schematically summarized in Figure 1.

Fresh porcine eyes were obtained from a local slaughterhouse. On arrival to the laboratory, eyes were washed in PBS and the corneal limbus was carefully dissected using sterile scissors. Limbi contained 2–3 mm of sclera and 3–4 mm of cornea. Limbal rings were washed thoroughly in PBS with a mixture of antibiotics and antimycotics containing penicillin (1000 U/mL), streptomycin (1000 µg/mL), and amphotericin B (2.5 µg/mL) (Merck, Darmstadt, Germany), and the rest of the uvea, retina, iris, and ciliary body were removed with forceps. The rings were then sectioned into fragments approximately 1 cm in length consisting of the full-thickness limbus (FL). Parts of these fragments were then sectioned in two halves using a surgical blade to separate the anterior part of the limbus (the most superficial) from the posterior part (the most profound). Only the anterior half of the limbal fragments, corresponding to the half-thickness limbus (HL), was used.

Both the FL and HL were subjected to four decellularization protocols combining several types of detergents (to dissolve cell membranes), distilled water (to induce osmotic cell lysis), NaCl (to promote cell swelling), and enzymes (to remove nucleic acids) (all these components were purchased from Merck):

− Protocol P1: Double-distilled water (ddH_2_O) for 24 h; 0.1% sodium dodecyl sulphate (SDS) (3 incubations of 24 h each).
− Protocol P2: ddH_2_O for 24 h; 0.1% SDS for 24 h; wash in PBS; 1.5 M of NaCl (2 incubations of 24 h each).
− Protocol P3: ddH_2_O for 24 h; 0.1% SDS for 24 h; wash in PBS; 1% sodium deoxycholate (SDC) for 24 h; wash in PBS; 0.6% triton X-100 for 24 h; wash in PBS; 100 mg/L of DNAse and 20 mg/L of RNAse for 45 min.
− Protocol P4: ddH_2_O for 24 h; 0.1% SDS for 24 h; wash in PBS; 1% sodium deoxycholate (SDC) for 24 h; wash in PBS; 0.6% triton X-100 for 24 h; wash in PBS; 100 mg/L of DNAse and 20 mg/L of RNAse for 45 min; wash in PBS; 0.05% Trypsin for 1 h.

All detergents were dissolved in ddH_2_O. DNAse, RNAse, and trypsin were used at 37 °C. All incubations were performed with agitation. After decellularization, decellularized limbi (DLs) were washed 5 times in cold PBS (15 min each time) and stored at 4 °C.

To assess transparency, DLs were placed on a black background and photographed.

### 2.2. Evaluation of Decellularization Efficiency in DL

To determine the efficiency of the four decellularization protocols applied to the corneal limbus, DLs were analyzed using DNA quantification and 4′,6-diamidino2-phenylindole (DAPI) staining. To quantify residual DNA in DLs, tissues were trimmed and processed using the QIAamp DNA Mini Kit (Qiagen, Hilden, Germany). Isolated DNA was dissolved in water and quantified using a NanoDrop 2000 spectrophotometer (Thermo Fisher Scientific, Waltham, MA, USA). Results were then normalized with respect to the weight of dry tissue as previously reported [35,36], and 10 measurements were made per sample. To identify the presence of nuclei or nuclei remnants in DLs, each tissue was fixed in formalin and embedded in paraffin as described below, and tissue sections were obtained using a microtome. Sections were dewaxed, rehydrated, stained with DAPI, coverslipped, and examined with a Nikon Eclipse i90 fluorescent microscope.

### 2.3. Generation of Recellularized Limbal Substitutes by Tissue Engineering

The DLs showing the best results were further recellularized with two types of cells: the limbal epithelial cell line SIRC (Statens Seruminstitut Rabbit Cornea) and primary cell cultures of mesenchymal stem cells (MSCs) derived from the adipose tissue (hADSCs). SIRC was purchased from ATCC (ref: CCL-60), whereas hADSCs were obtained by the enzymatic digestion of small human adipose tissue biopsies, as previously reported [37]. Both cell types were cultured in Dulbecco’s modified Eagle’s medium (DMEM) supplemented with 10% fetal bovine serum and 1% antibiotics/antimycotics (all from Merck) using standard cell culture conditions.

To obtain recellularized limbi (RLs), DLs were first functionalized to increase the adhesiveness of the decellularized scaffold and promote cell attachment by incubating the tissues in fetal bovine serum for 24 h with slight agitation.

After functionalization, SIRC and hADSCs were trypsinized and carefully seeded on the surface of the DL (170,000 cells per cm^2^ of sample). To promote attachment, cells were resuspended in a minimal amount of medium (50 µL) and DLs were immobilized using agarose casts, as previously reported [38]. In order to induce epithelial differentiation of both cell types seeded on the RL, these tissues were cultured for 21 days in EM epithelial differentiation medium containing epithelial growth and differentiation factors, as previously described [39]. The EM medium consisted of a mixture of 150 mL of HAM-F12, 300 mL of DMEM, 50 mL of fetal bovine serum, 1% antibiotics/antimycotics, 24 μg/mL of adenine, 5 μg/mL of insulin, 1.3 ng/mL of triiodothyronine, 0.4 μg/mL of hydrocortisone, and 10 ng/mL of EGF (epidermal growth factor) (all of them, from Merck).

Preliminary transmittance analysis was carried out on RLs and controls using a SmartSpec 3000 spectrophotometer (Bio-Rad, Hercules, CA, USA). Each sample was analyzed at three wavelengths (400, 550, and 700 nm) using three replicates, and average values were obtained.

### 2.4. Histological Analyses of DL and RL

Control porcine and human native limbi, and DLs and RLs were fixed in 4% neutral buffered formaldehyde, dehydrated in increasing concentrations of ethanol, cleared in xylene, and embedded in paraffin following routine protocols. In addition, 5 μm sections were obtained with a microtome, mounted on glass slides, dewaxed, and rehydrated with an ethanol series.

To evaluate tissue morphology and structure, sections were stained with hematoxylin-eosin (HE) (Panreac AppliChem, Barcelona, Spain). The structure and composition of the tissue extracellular matrix (ECM) were evaluated by identifying collagen fibers and proteoglycans using picrosirius red (PSR) and alcian blue (AB) histochemistry, as previously reported [36,40] (reagents from Panreac AppliChem).

In order to identify specific components of the epithelial and basement membrane layers of RLs, controls and RLs were subjected to immunohistochemistry for p63, pancytokeratin, crystallin Z (CRY-Z), laminin, and collagen IV. In brief, tissue sections were subjected to antigen retrieval with pH 8 EDTA buffer (25 min at 95 °C) for p63, pancytokeratin, and collagen IV or with pH 6 citrate buffer (25 min at 95 °C) for CRY-Z and laminin, and endogenous peroxidase was quenched with H_2_O_2_. Then, samples were preincubated in a blocking solution containing horse serum and incubated with the following primary antibodies: anti-p63 (Master Diagnostica, Granada, Spain, prediluted), anti-pancytokeratin (Master Diagnostica, prediluted), anti-CRY-Z (Abcam, Cambridge, UK, dilution 1:250), anti-laminin (Abcam, dilution 1:200), and anti-collagen IV (Master Diagnostica, prediluted). After washing in PBS, tissues were incubated in secondary anti-mouse or anti-rabbit antibodies labeled with peroxidase (ImmPRESS reagent kit, Vector Laboratories; Burlingame, CA, USA, prediluted), washed in PBS, and incubated with diaminobenzidine (DAB) (Vector Laboratories). In all cases, positive and negative control tissues were used, with negative controls corresponding to tissue sections subjected to the same protocol, except that the primary antibody was replaced by PBS to show the negative staining signal. Samples were then counterstained with Harry’s hematoxylin and coverslipped.

### 2.5. Quantitative Analysis and Statistics

Stained tissues were analyzed with an Eclipse 90i microscope (Nikon, Tokyo, Japan), and images were obtained using the same conditions (magnification, exposure time, contrast, etc.) for all samples stained with the same method to allow signal quantification. White light was used to analyze all samples, and polarized light microscopy was used to evaluate DL tissues stained with PSR.

For PSR and AB histochemistry, the staining signal intensity and area fraction were quantified using the ImageJ software (National Institutes of Health, Bethesda, MD, USA), as previously reported [41]. Briefly, each histological image was analyzed by randomly selecting 10 points (for intensity) and 10 square areas (for area fraction), and both the signal intensity and the area occupied by the positive staining signal were calculated by the program, the background signal was subtracted, and averages were obtained for each type of sample. Results obtained for each sample were statistically compared with controls using the Mann–Whitney tests with the RealStatistics software (Dr. Charles Zaiontz, Purdue University, West Lafayette, IN, USA).

For the immunohistochemical analyses, results were semiquantitatively categorized as strongly positive signal (+++), positive signal (++), slightly positive signal (+), or negative signal (−), as previously reported [39].

## 3. Results

### 3.1. Decellularization Efficiency of the Different Protocols Applied to the Porcine Limbus

**Figure 2:**
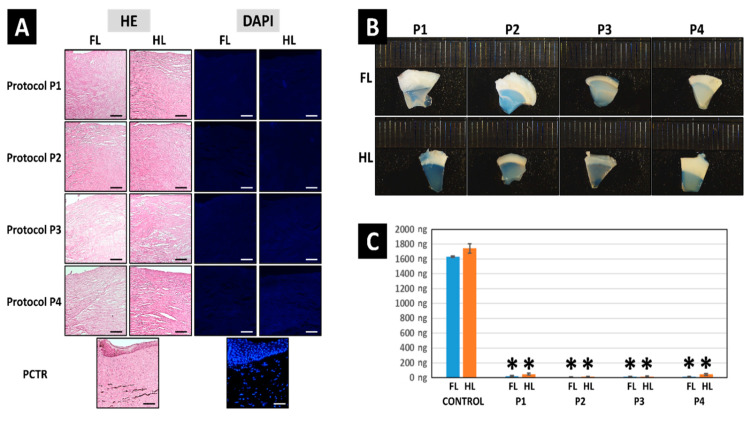
Analysis of native porcine limbus (PCTR) and decellularized limbi using four different decellularization methods (P1 to P4). Each decellularization protocol was applied to the full-thickness limbus (FL) and the half-thickness limbus (HL). (**A**) Histological analysis using hematoxylin-eosin (HE) and DAPI. (**B**) Macroscopical images showing transparency levels of each DL on a black scale in millimeters. (**C**) Quantification of residual DNA (in ng of DNA per mg of dry weight of tissue) in controls and DL. Asterisks (*) represent statistically significant differences with both the FL and the HL controls (*p <* 0.05). Nonsignificant differences were found among the different samples decellularized with P1, P2, P3, and P4. Scale bars: 50 μm.

Analysis of the different decellularization protocols studied in this work revealed that the four protocols were able to efficiently decellularize the porcine corneal limbus. First, the efficiency of the decellularization process was evaluated by quantification of the residual DNA present in each type of sample. Results showed very high DNA content in control limbi (1742.58 ± 62.06 ng of DNA per mg of dry weight of tissue), whereas DL tissues subjected to decellularization had very low amounts of DNA, with all protocols showing less than 50 ng of DNA per mg of dry weight of tissue for both the FL and HL, thus fulfilling the requirements for decellularized tissues [35] (Figure 2). Differences with control FL and HL were statistically significant for all groups, but comparisons among the different types of decellularized tissues showed nonsignificant differences.

In order to evaluate the decellularization efficiency at the histological level (Figure 2), controls and DLs decellularized with each protocol were analyzed histologically using HE staining. As shown in Figure 1, native control limbi showed abundant cells at the epithelial and stromal layers of the tissue. However, the use of the four decellularization methods evaluated here resulted in a complete absence of detectable cells or cell debris in all DLs, for both the HE and DAPI staining methods, with no differences among samples, suggesting that the four methods described here were fully efficient, although the typical pocket-like structures found in the limbal area were not detected in DLs.

Strikingly, we found that tissues decellularized with protocols P1, followed by P2, showed the most appropriate results in terms of transparency, especially when HLs were used. In contrast, P3 and P4 resulted in an important alteration of corneal transparency (Figure 2).

### 3.2. Histochemical Analysis of ECM Components Preservation in Decellularized Limbi

**Figure 3:**
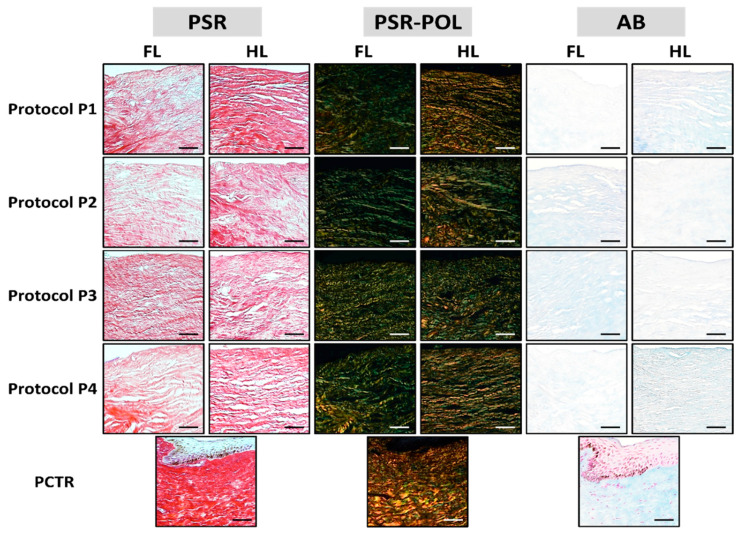
Histochemical analysis of native porcine limbus (PCTR) and decellularized limbi using four different decellularization methods (P1 to P4). Each decellularization protocol was applied to the full-thickness limbus (FL) and the half-thickness limbus (HL). PSR: picrosirius red, PSR-POL: polarized-light picrosirius red, AB: alcian blue. Scale bars: 50 μm.

**Figure 4:**
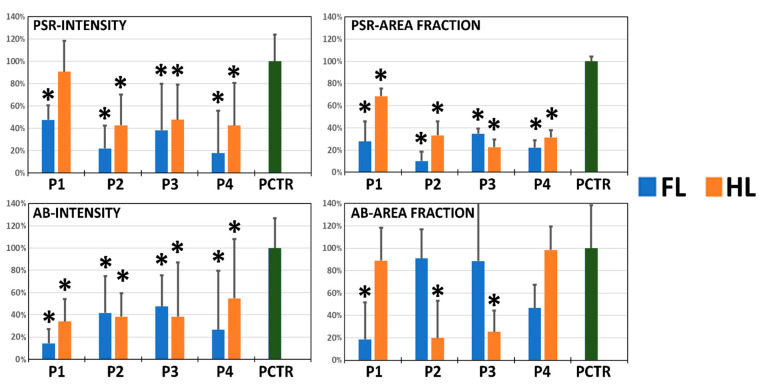
Quantitative analysis of the picrosirius red (PSR) and alcian blue (AB) staining intensity and area fraction of decellularized limbi (DLs). Four decellularization protocols (P1 to P4) were applied to the full-thickness limbus (FL, blue bars) and the half-thickness limbus (HL, orange bars). Results are shown as average values normalized with respect to the native porcine limbus used as control (PCTR, green bars), which is considered as 100%, with error bars corresponding to standard deviations. Asterisks (*) represent statistically significant differences with the native porcine limbus used as control (*p <* 0.05).

The effects of each decellularization protocol on the structure and composition of the tissue ECM were evaluated using PSR and AB (Figure 3 and Figure 4). As expected, we first found that native control limbi showed high PSR staining intensity and area fraction, suggesting that a high number of collagen fibers were present in these tissues. Then, the analysis of DLs revealed a significant decrease in PSR intensity and area fraction in all samples (*p <* 0.05), except for HLs treated with the P1 protocol, which were comparable to controls for PSR staining intensity but were significantly lower than controls for the area fraction occupied by collagen fibers.

When polarized light was applied, we found that control corneas showed several types of properly oriented collagen fibers, with a mixture of red, orange, yellow, and green fibers. However, DLs tended to show a decrease in red and orange colors, especially in FLs and in HLs treated with protocol P3, suggesting a decrease in thick, mature fibers and an alteration of fiber alignment and orientation in these samples, as previously suggested [42].

Analysis of tissue proteoglycans using AB staining showed a significant decrease in the staining signal intensity in all samples, as compared to control native tissues (*p <* 0.05). However, when the area fraction corresponding to an AB-positive signal was analyzed, we found a significant decrease in FLs decellularized with P1 and in HLs treated with P2 and P3, with the rest of samples being comparable to controls (*p >* 0.05).

On the other hand, our preliminary analysis of the transparency of RLs showed that the average transmittance of these tissues ranged between 31.49 ± 9.14% of the transmittance of HCTR found in RLs recellularized with hADSCs at 21 days of follow-up and 102.44 ± 32.06% for limbi recellularized with SIRC at day 21 (Supplementary Table S1).

### 3.3. Histological Analysis of Recellularized Limbi

**Figure 5:**
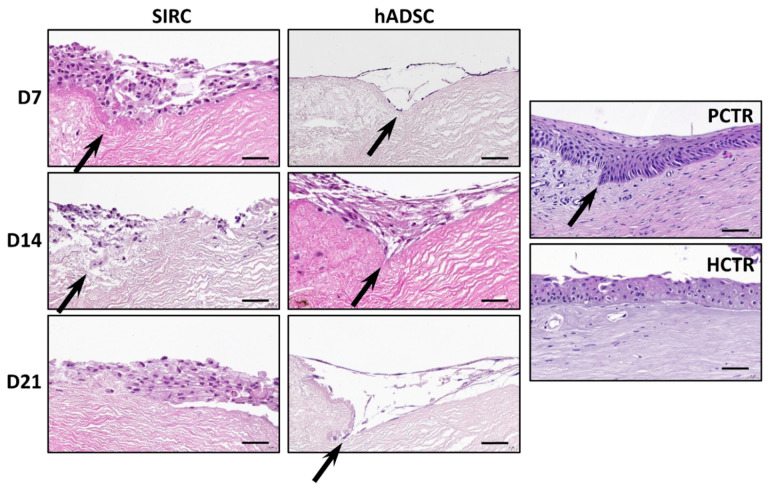
Histological analysis of native controls and RLs recellularized with SIRC epithelial cells and hADSCs, at days 7 (D7), 14 (D14), and 21 (D21) of follow-up using hematoxylin-eosin staining (HE). PCTR: Native porcine limbus used as control. HCTR: Native human limbus used as control. Pocket-like structures found in some of the images have been highlighted with arrows. Scale bars: 50 µm.

In the present work, we used DLs decellularized with protocol P1 applied to HLs, as this method allowed an efficient decellularization with the best results in terms of ECM preservation. When these tissues were recellularized with SIRC rabbit cornea epithelial cells, we found that cells tended to attach to the DL surface, forming a multilayered cell stratum, and tended to allocate in the pocket-like structures found in DLs (Figure 5). In addition, we found that the number of cells in each RL was high from the first analysis time at day 7, with very few changes at days 14 and 21. Cells showed several intercellular spaces at days 7 and 14, but not at day 21, when cells became more densely packed, although the well-organized structure of the native cornea epithelium was not reached.

Analysis of RLs containing hADSCs revealed that this type of cell was also able to attach to the tissue surface, but the number of cells was low at day 7, with few cell layers, and increased at day 14, with several cell layers. Interestingly, some of the cells became detached from the decellularized scaffold at day 21. As for the SIRC cells, abundant intercellular spaces were found among hADSCs, and differed from the fine structure of the control tissues. Interestingly, the morphology of the hADSCs grown on the surface of the RL was elongated and spindle-shaped, whereas SIRC displayed a more rounded or polygonal shape.

### 3.4. Evaluation of Limbal Cell Markers in Recellularized Limbi

**Figure 6:**
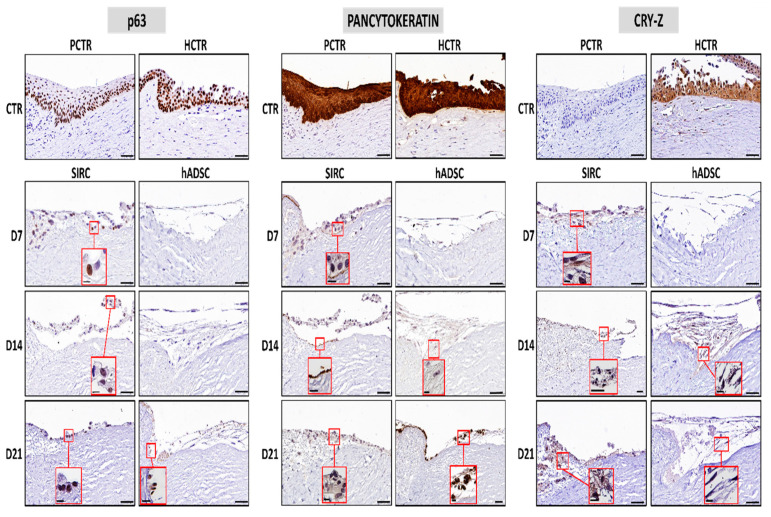
Immunohistochemical analysis of the corneal epithelial cell markers p63, pancytokeratin, and crystallin Z (CRY-Z) in RLs recellularized with SIRC cells and hADSCs at days 7 (D7), 14 (D14), and 21 (D21) of follow-up, porcine native limbus (PCTR), and human native limbus (HCTR). Insets correspond to higher-magnification images of cells showing the expression of each analyzed marker in the RL tissues. Scale bars: 50 µm for large images and 10 µm for the insets.

In the first place, we analyzed the expression of the limbal stem cell marker p63 in controls and RLs (Figure 6). As expected, epithelial cells found in control limbi were strongly positive (+++), especially in the human limbus and in the basal layer of the porcine limbus. When the RLs were analyzed, we found that tissues recellularized with SIRC epithelial cells showed positive p63 expression (++) from day 7 to day 21, although at a lower level than controls. However, hADSCs showed negative p63 expression (−) at days 7 and 14, and became positive (++) at day 21. Then, we assessed the expression of pancytokeratin in each type of sample, and we found a strongly positive signal (+++) in human and porcine control limbi, and a positive expression in RLs containing SIRC cells at 7, 14, and 21 days of follow-up. In addition, the expression was negative (−) in RLs recellularized with hADSCs kept ex vivo for 7 days, slightly positive at day 14, and became positive at day 21. Finally, our analysis of CRY-Z proteins revealed that human epithelial cells were strongly positive, although porcine cornea cells were negative for this marker. RLs containing SIRC were positive at the three time periods analyzed here, whereas RLs containing hADSCs were negative at day 7, and slightly positive at days 14 and 21 of follow-up ex vivo.

### 3.5. Immunohistochemical Analysis of Basement Membrane Components in Recellularized Limbi

**Figure 7:**
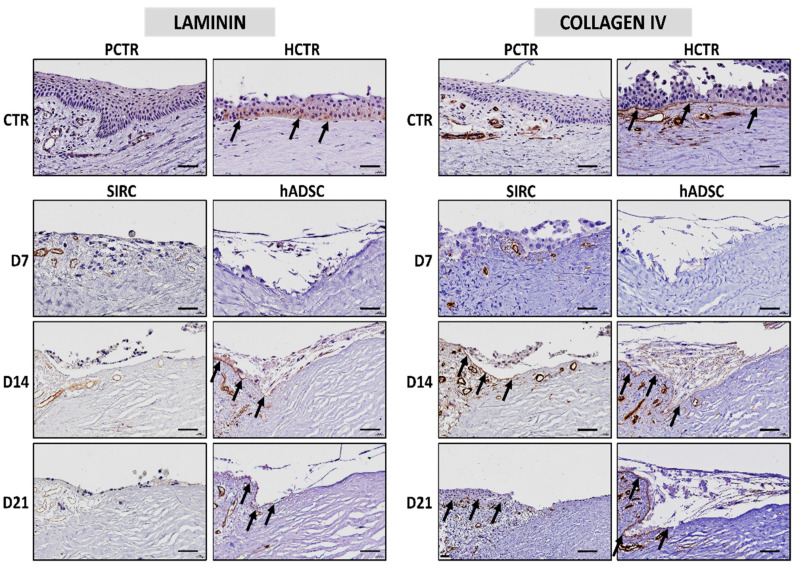
Immunohistochemical analysis of the basement membrane markers laminin and collagen IV in RLs recellularized with SIRC cells and hADSCs at days 7 (D7), 14 (D14), and 21 (D21) of follow-up, porcine native limbus (PCTR), and human native limbus (HCTR). Illustrative areas of the basement membrane stained by the immunohistochemical procedure are highlighted with arrows. Scale bars: 50 µm.

Two of the main components of the basement membrane—laminin and collagen IV—were analyzed by immunohistochemistry (Figure 7). In this regard, our results demonstrated that the human native cornea expressed both proteins at the basement membrane of the epithelial cells, although the porcine cornea was negative for the two markers analyzed here. RLs containing SIRC cells were negative for laminin at all follow-up times, but were positive for collagen IV at days 14 and 21, being negative at day 7. However, RLs generated with hADSCs were negative for laminin and collagen IV at day 7 and became positive at days 14 and 21 for both markers. As expected, blood vessels found at the limbal tissue showed a positive staining signal for both laminin and collagen IV.

### 3.6. Histochemical Analysis of ECM Components in Recellularized Limbi

**Figure 8:**
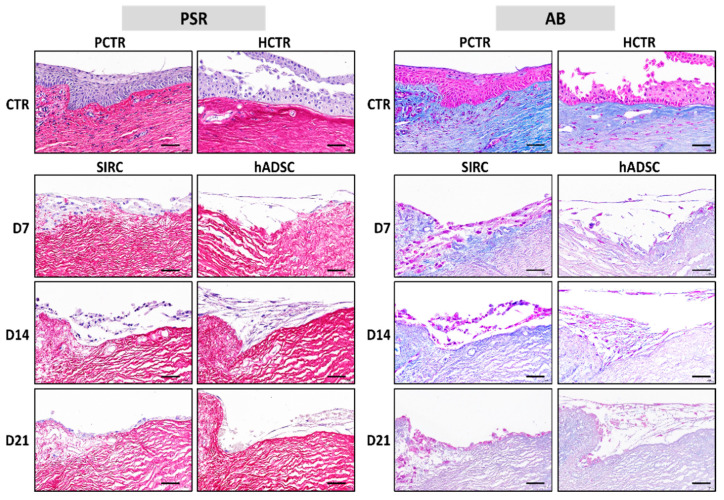
Histochemical analysis of native porcine limbus (PCTR), native human limbus (HCTR), and limbi recellularized with SIRC cells and hADSCs evaluated at days 7 (D7), 14 (D14), and 21 (D21) of follow-up. PSR: picrosirius red, AB: alcian blue. Scale bars: 50 μm.

**Figure 9:**
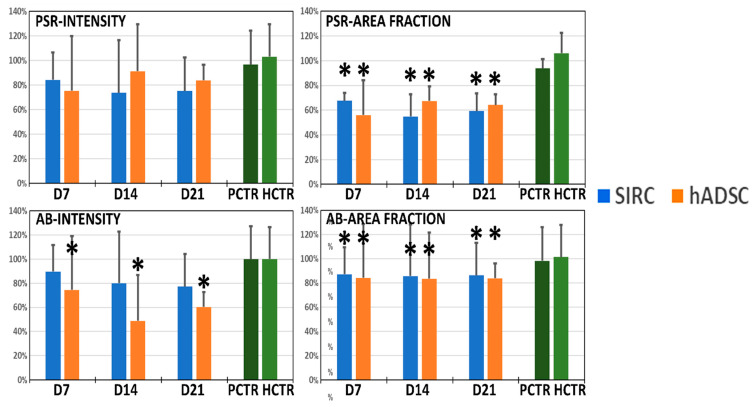
Quantitative analysis of the picrosirius red (PSR) and alcian blue (AB) staining intensity and area fraction of recellularized limbi (RLs). Results are shown as average values normalized with respect to the native limbi used as controls and shown in green (PCTR and HCTR), whose mean is considered as 100%, with error bars corresponding to standard deviations. Asterisks (*) represent statistically significant differences with both controls (PCTR and HCTR) (*p <* 0.05).

Once recellularized, two major ECM components were analyzed in RL samples using PSR and AB (Figure 8 and Figure 9). When the staining intensity was analyzed in samples stained with PSR, we found that the highest intensity corresponded to the native limbus controls. However, differences between controls and all types of RLs were nonsignificant at 7, 14, and 21 days (*p >* 0.05). However, we found that all RLs had lower PSR area fractions than native controls did at the three times, with significant differences between the native limbi and all types of RLs.

Analysis of ECM proteoglycans using AB histochemistry first revealed that the lowest staining intensity corresponded to RLs containing hADSCs, with statistically significant differences with native limbi for the three time periods analyzed here. In contrast, RLs generated with SIRC showed nonsignificant differences with controls at all times. Finally, we found that the area occupied by AB-positive staining was significantly lower in all types of RLs than in native controls at all times, with very few differences among RL samples and times.

## 4. Discussion

LSCD is a severe condition causing cornea opacification, conjunctival pannus, and blindness that can be secondary to chemical or thermal injuries, autoimmune diseases such as the Stevens–Johnson syndrome, mucous membrane pemphigoid, and hereditary diseases such as aniridia [43]. Current treatments are challenging, especially in cases with structural damage of the limbal area, which is not a candidate to cell therapy. In fact, maintenance of the crypt-like structures in which limbal stem cells reside is fundamental for these cells to survive and exert their function in the limbus, and structural alterations of this niche would lead to stem cell death [6].

In the present preliminary work, we generated several types of limbal substitutes that could be used in the future to replace the damaged limbus using both a corneal and an extracorneal cell source. Although the present report is a preliminary work, our results suggest that these bioartificial limbi display several similarities with the native limbus and, thus, could be potentially useful for the treatment of LSCD.

In the first place, we evaluated several decellularization protocols applied to the native porcine limbus. Decellularization of native organs allows the obtaining of biological scaffolds composed of natural extracellular matrix (ECM) that can be used in regenerative medicine for tissue and organ replacement [35]. Compared with strategies based on scaffolds generated de novo such as fibrin, collagen, or agarose applied to cornea tissue engineering [20,44], decellularization offers the possibility of obtaining a scaffold containing the Vogt palisades and crypt-like structures that are required for a proper limbal function [30]. In addition, previous works have demonstrated that the porcine limbus is structurally similar to the human limbus [45].

Xenografts obtained by the decellularization of animal tissues can be used to reproduce human tissues and organs in the laboratory [46]. Compared with human tissues, xenografts are easily available and accessible, and can be obtained with very few ethical concerns. However, xenogeneic scaffolds may not be able to fully reproduce the structure and biochemical composition of the human tissues, and the expression of relevant antigenic components should be controlled before clinical use, as antigenicity could hinder the use of xenografts in regenerative medicine [47].

Numerous protocols have been described to date for the decellularization of different types of corneal xenografts [24]. However, very little information is available on decellularization of the corneal limbus. In a preliminary work, we demonstrated previously that the porcine cornea can be decellularized in toto, including the limbus, using SDS detergents [25]. Then, Huang et al. used a combined protocol using a mixture of salts, enzymes, and SDC and demonstrated that the porcine limbus could be decellularized and then recellularized with cultured cells [48]. Very recently, Isidan et al. compared several methods applied to the whole porcine cornea and confirmed our preliminary results suggesting that SDS is the most effective decellularization agent for the whole cornea [28]. Based on the protocols described in all these previous reports, we selected four decellularization methods that were evaluated in the present work. In general, these methods were based on the use of the anionic detergents SDS and SDC, the nonionic surfactant Triton X-100, hypertonic NaCl, and trypsin digestion.

Previous reports have suggested that decellularization of the whole cornea is challenging, and the efficiency of the decellularization protocols may be reduced when the whole tissue is subjected to decellularization [28]. For this reason, we evaluated each decellularization protocol on both the full-thickness limbus and the half-thickness limbus. Our results showed that all protocols were efficient on both the FL and the HL tissues, suggesting that the four protocols evaluated here were fully successful and are appropriate for decellularization of the porcine limbus. However, protocol P1, which is based on the use of SDS detergent, was able to preserve the limbus ECM components and transparency more efficiently than other protocols could, especially when HLs were used. Although differences were found with control native corneas, and the crypt-like structures were not detectable, probably due to tissue swelling, the collagen staining intensity and the proteoglycans area fraction were comparable with controls. For these reasons, and due to the fact that protocol P1 is one of the simplest decellularization protocols, we could recommend this protocol applied to HLs for porcine limbus decellularization. This is in agreement with previous results obtained by our group [25] and by other research groups [28]. However, additional analyses based on biochemical characterization of the decellularized limbi should be performed to confirm these findings. In addition, our analysis of transparency was very preliminary, and in-depth analyses able to quantify the transmittance of each DL with higher accuracy should be performed before and after glycerol treatment—to reduce swelling—as suggested [49].

An important issue related to decellularized tissues is biomechanical behavior. In general, it is well known that the biomechanical properties of tissues are important variables affecting tissue function and cell mechanotransduction [50]. However, the decellularization process may significantly alter the structure of the tissue ECM and, thus, the biomechanical properties of the decellularized scaffolds, which could alter the phenotype, proliferation capability, and differentiation potential of the cells cultured on this scaffold and modify cell behavior and tissue regeneration [51]. In fact, it has been demonstrated that decellularized corneal xenografts vary their biomechanical properties after decellularization [52]. Therefore, a thorough analysis of the biomechanical properties of the DL generated in the present work is in need.

Once decellularized, DLs should be repopulated with limbal stem cells for clinical use. Recellularization is also challenging, as not all cell types are able to grow and differentiate on decellularized scaffolds. In the present work, we assessed two different types of cells for limbal recellularization: corneal epithelial cells and extracorneal cells with differentiation potential (hADSCs). The use of alternative cell sources was previously suggested by several researchers, who demonstrated that hADSCs have intrinsic potential to differentiate into several cornea cell phenotypes both ex vivo and in vivo [33,34,53]. First, different types of MSCs were differentiated into stromal keratocytes using conditioning media [34], suggesting that these cells could be used to support epithelial cell growth and differentiation. However, these cells were also demonstrated to have a differentiation potential into cornea epithelial cells [33,34,53], which supports their use as alternative cell sources in cornea and limbus recellularization. In general, our results suggest that both types of cells were able to attach to this scaffold and showed several markers of cell differentiation on the decellularized biomaterials. Interestingly, the morphology of each cell type was different, and hADSCs showed the typical structure of MSCs, and abundant intercellular spaces, at day 21. In general, these findings support the preferential use of SIRC over hADSCs, as SIRC cells are specifically committed to the limbal epithelial cell lineage, whereas hADSCs correspond to the undifferentiated phenotype that is typical of MSCs [54].

Although further research should determine the role of serum functionalization, the fact that cells were able to adhere to the decellularized scaffold could be related to the functionalization step applied to the DL. Previous reports have shown that most biomaterials lack specific signals that are necessary for cell differentiation and function, and surface functionalization with serum proteins could contribute to mimic the in vivo scenario and improve biomaterial functionality [55]. A possible concern of tissue functionalization with serum is the possibility of inducing cells to differentiate to noncorneal cell lineages, such as the vascular phenotype. Future studies should determine if functionalization is necessary and if alternative methods can be applied to DLs.

To determine the feasibility of the scaffolds generated in this work to support limbal cell differentiation, we analyzed the expression of several markers of epithelial differentiation. In general, our results obtained ex vivo suggest that none of the cell types were able to fully differentiate and mature on the scaffold, although partial signs of epithelial differentiation were found. Concretely, SIRC cells were able to express the epithelial markers p63, pancytokeratin, and CRY-Z from the beginning, with no time-dependent differences. This is in agreement with the limbal epithelial stem cell nature of these cells and their intrinsic differentiation status [56]. However, hADSCs were initially negative for these three markers, as is the case of all types of human MSCs, but became positive or slightly positive for the three epithelial markers after 21 days of ex vivo differentiation induction using conditioning media. These results confirm the differentiation capability of hADSCs to the epithelial cell lineage under certain circumstances, as previously suggested [57,58]. Previous results published by our group demonstrated that these cells, which can be harvested autologously, can be differentiated ex vivo using conditioning media, although differentiation is not complete ex vivo and the in vivo environment is required for terminal differentiation [58]. Interestingly, hADSCs were already used to efficiently recellularize acellular scaffolds obtained from human corneas [59]. Future studies should be carried out on animal models to determine if these cells are able to fully differentiate into epithelial cells upon in vivo induction, as demonstrated for the skin and oral mucosa [58,60]. Moreover, additional research is in need to fully characterize the cells grown on the decellularized scaffolds to determine their exact phenotype. Specifically, immunostaining with the Ki-67 proliferation marker and labeling with BrdU should determine the proliferation potential of these cells, whereas co-staining with limbal stem cell markers such as ABCG2, p63, and cytokeratin 15 should demonstrate their stem cell identity [61].

One of the main factors influencing epithelial cell attachment is the basement membrane. Evaluation of this structure showed that two of its major components—laminin and collagen IV—were detectable in RLs from day 14 onward, suggesting that an incipient basement membrane was formed between the scaffold and the cells seeded on top. The fact that RLs containing SIRC were negative for laminin could be explained by the fact that the anti-laminin antibody used in this work was specific anti-human.

In addition, the quantification of two key components of the limbus ECM revealed that RLs containing SIRC cells had adequate collagen fibers and proteoglycans—in terms of staining intensity—although the area fraction was not comparable to controls. The fact that SIRC cells are already committed to the limbal phenotype, whereas hADSCs are much more undifferentiated, may explain these findings. Despite its effect on collagen and proteoglycans quantification likely being very low or negligible, it is also possible that ECM components found in RLs may be affected by tissue functionalization with serum.

The present work has several limitations. The first one is the need of carrying out additional analyses, such as a biochemical analysis, to confirm the histological, histochemical, and immunohistochemical results showed here, as well as extensive analysis of tissue transparency after decellularization and recellularization. Furthermore, DLs should be analyzed using transmission electron microscopy techniques to determine if the limbal crypts are intact after the decellularization process. In addition, DLs should be recellularized with human primary limbal stem cells, and the expression of relevant stem cell markers should be assessed in these cells such as specific limbal stem cell markers and cell proliferation markers, to determine the real potential of the decellularized scaffolds. Future analyses should address all these issues.

In summary, the preliminary results obtained in the present work demonstrated that the porcine cornea limbus can be efficiently decellularized using the protocols described here, and that recellularization with epithelial or mesenchymal cells allows the successful generation of RLs with potential clinical usefulness. Future experiments in animal models should determine the in vivo usefulness of these limbal substitutes. Among their possible clinical applications, the limbal substitutes described here could be used as advanced therapies and medicinal products in patients with severe structural alteration of the limbus and loss of the micro-niche of the limbal stem cells.

## 5. Patents

MCM and MA are coauthors of patent application number PCT/ES2020/070168, “decellularized limbus”.

## Data Availability

The data presented in this study are available on request from the corresponding authors.

## Supplementary Materials

The following are available online at https://www.mdpi.com/article/10.3390/pharmaceutics13101718/s1, Table S1: Preliminary analysis of light transmittance at three different wavelengths (400, 550 and 700 nm) as determined by spectrophotometric analysis of human native limbus (HCTR) and RL decellularized with SIRC and hADSC cells at days 7, 14 and 21 of follow-up. Values correspond to percentages of transmittance using the values obtained in HCTR as reference (100% transmittance).
