# Supplementary material for: Generation of a Biomimetic Substitute of the Corneal Limbus Using Decellularized Scaffolds
Source: Pharmaceutics. 2021 Oct 17;13(10):1718. doi: 10.3390/pharmaceutics13101718 (PMC8541096; doi:10.3390/pharmaceutics13101718)
Supplement: Supplementary file 1 [file pharmaceutics-13-01718-s001.zip › pharmaceutics-1372919-supplementary.pdf]

pharmaceutics

Supplementary Materials: Generation of a Biomimetic Substitute of the Corneal Limbus Using Decellularized Scaffolds

David Sánchez-Porras, Manuel Caro-Magdaleno, Carmen González-Gallardo, Óscar Darío García-García, Ingrid Garzón, Víctor Carriel, Fernando Campos and Miguel Alaminos

Table S1. Preliminary analysis of light transmittance at three different wavelengths (400, 550 and 700 nm) as determined by spectrophotometric analysis of human native limbus (HCTR) and RL decellularized with SIRC and hADSC cells at days 7, 14 and 21 of follow-up. Values correspond to percentages of transmittance using the values obtained in HCTR as reference (100% transmittance).

	400 nm	550 nm	700 nm	Average
HCTR	100±0.03	100±2.22	100±3.04	100±0
RL-SIRC-D7	70.76±0.07	50.66±0.47	64.53±0.76	61.98±10.29
RL-SIRC-D14	50.5±0.08	50.31±1.09	72.84±2.16	57.88±12.96
RL-SIRC-D21	138.51±0.18	77.22±0.64	91.58±0.34	102.44±32.06
RL-hADSC-D7	121.13±0.07	66.94±0.76	82.88±1.63	90.32±27.85
RL-hADSC -D14	15.73±0	31.68±0.03	61.9±0.15	36.44±23.45
RL-hADSC -D21	24.51±0.02	28.12±0.05	41.83±0.31	31.49±9.14
